# Stability, accuracy, and clinical performance of enzymatic total CO₂ measurement: Evaluation of the Snibe and Roche assays

**DOI:** 10.1371/journal.pone.0334228

**Published:** 2025-10-10

**Authors:** Shuai Wei, Zhonggang Fang, Min Shi, Qunxian Zhang, Qiang Huang, Gao Chen, Zhenlei Wei, Chen Sun, Haolin Wei, Tinghua Li, Chen Lan

**Affiliations:** 1 Department of Clinical Laboratory, The People’s Hospital of Hechi, Hechi, Guangxi, P.R. China; 2 Research & Development Department, Shenzhen New Industries Biomedical Engineering Co., Ltd. (Snibe), Shenzhen, P.R. China; 3 Modern Industrial College of Biomedicine and Great Health, Youjiang Medical University for Nationalities, Baise, Guangxi, P.R. China; University of Toronto Department of Laboratory Medicine and Pathobiology, CANADA

## Abstract

**Objectives:**

To evaluate the stability, accuracy, and clinical performance of the Snibe CO₂ assay compared with the Roche CO₂ reagent and to investigate factors affecting total CO₂ measurements in different clinical conditions.

**Methods:**

Total CO₂ was measured in patient samples using enzymatic assays. Stability was assessed over time, and CO₂ loss was examined in different blood collection tubes. Method comparison was conducted between the Snibe and the Roche CO₂ assays in various diseases.

**Results:**

The Snibe CO₂ assay showed excellent linearity (r = 0.9997) and maintained stability for 42 days without recalibration, with control material deviations within ±5% of the target. CO₂ loss was observed in uncapped non-anticoagulant tubes (24.0% decline over 48 hours), but was less in EP tubes (9.4% decline). Significant differences of median CO₂ measurements were observed in only the renal group (Snibe: 13.35 mmol/L, Roche: 12.80 mmol/L) and the cardiovascular disease group (Snibe: 25.90 mmol/L, Roche: 26.40 mmol/L) versus the healthy group (Snibe: 22.70 mmol/L, Roche: 22.90 mmol/L) for both the Snibe and Roche assays.

**Conclusions:**

The Snibe CO₂ assay demonstrated superior stability and comparable accuracy compared with the Roche reagent. Preanalytical CO₂ loss remains a critical issue, emphasizing the need for standardized sample handling. Given the correlation between total CO₂ and bicarbonate levels, accurate measurement is critical for diagnosing metabolic disorders. Laboratories should establish protocols to minimize errors and ensure reliable acid-base assessment in clinical practice.

## 1. Introduction

Carbon dioxide (CO₂) is a critical metabolic byproduct generated by cellular respiration [1]. In the human body, the majority of CO₂ is transported in the blood in the form of bicarbonate (HCO₃⁻), accounting for approximately 95% of the total CO₂ concentration in plasma [2]. The remaining CO₂ is present in its dissolved form, as carbonic acid (H₂CO₃), and in small amounts as carbamino compounds bound to proteins, particularly hemoglobin [3]. This transformation of CO₂ into bicarbonate ions plays an essential role in maintaining acid-base balance [4]. As the body’s primary buffering system, bicarbonate works in tandem with the respiratory and renal systems to regulate blood pH, ensuring proper physiological function across various systems [5]. Total carbon dioxide (TCO₂) measurement is a key diagnostic tool in clinical chemistry, routinely used to assess and monitor metabolic and respiratory disturbances [6]. Accurate TCO₂ measurements are fundamental for diagnosing acid-base disorders, determining the underlying causes, and monitoring the effectiveness of therapeutic interventions [7]. In the context of respiratory function, CO₂ levels provide insight into both ventilatory status and the body’s compensatory mechanisms in response to fluctuations in pH [8].

Elevated TCO₂ levels are commonly associated with conditions of alkalosis, either metabolic or respiratory [4]. Metabolic alkalosis can result from excessive gastric acid loss due to pyloric obstruction, upper small intestine obstruction, hypokalemia, and excessive intake of alkaline substances like antacids or bicarbonate [9]. Respiratory alkalosis, on the other hand, arises from increased excretion of CO₂ due to hyperventilation such as in anxiety or high altitude [10]. Conversely, decreased TCO₂ levels could indicate metabolic acidosis associated with conditions like diabetic ketoacidosis [11] or renal failure [12]. On the other hand, respiratory acidosis results from impaired CO₂ elimination from the lungs, which can occur in respiratory muscle paralysis, airway obstruction related to diseases like chronic obstructive pulmonary disease (COPD), or acute lung diseases like pneumonia and acute respiratory distress syndrome (ARDS) [10]. Therefore, TCO2 measurement is widely used in the clinical evaluation of these acid-base disorders. As acid-base disturbances are prevailing in critically ill patients, who tend to have rapid changes in CO₂, and are associated with increased mortality, accurate measurement of TCO₂ is a determining factor of the patients’ clinical outcomes.

Traditional methods of measuring TCO₂ include the manual titration method, which is time consuming and susceptible to interferences from temperature, pH and operators [13]. Ion-selective electrodes (ISE) and enzymatic methods emerged as more reliable alternatives. The ISE method, although widely adopted in clinical laboratories, is sensitive to variability in pH, ionic strength, electrode selectivity, and interference from other ions [14,15]. The enzymatic assays are compatible with automated biochemical analyzers and allow for high-throughput, rapid, and accurate measurements, making them well-suited for routine clinical use [16]. Despite these advantages, enzymatic TCO₂ assays still face the challenge of reagent instability. As ambient CO₂ is abundant, the regents would be rapidly consumed by the absorption of ambient CO₂ once the container is opened [17]. This issue would cause large variability in between-day quality controls and falsely reduced results due to insufficient substrates, a problem that would be more pronounced in smaller laboratories which use reagents less frequently.

The Snibe CO₂ reagent is a single-liquid enzymatic assay specifically designed to address these stability concerns. By inhibiting enzyme during cold storage through the adjustment of substrate, pH, buffering system and the addition of activation reagent, the Snibe assay has accomplished minimized loss of substrate and demonstrated significantly improved reagent stability. To facilitate the clinical application of such a promising assay, this study evaluated the analytical performance of the Snibe CO₂ assay, with a focus on its accuracy, calibration stability, and on-board stability, in comparison to a similar kit manufactured by Roche. Additionally, the study investigated the impact of various pre-analytical conditions on serum CO₂ sample stability, providing crucial guidance for routine clinical use of this assay to ensure that reliable results could be obtained in everyday practice.

## 2. Materials and methods

### 2.1. Patients and samples

Between 27th August 2024 and 8th January 2025, a total of 320 patients were enrolled in this study. Patients≥18 years old were included if they had symptoms related to metabolic alkalosis (hypokalemia or hyperadrenocorticism), respiratory acidosis (airway obstruction, severe emphysema, bronchiectasis or pulmonary edema), metabolic acidosis (diabetic ketoacidosis, uremia or severe diarrhea) and respiratory alkalosis (tachypnea or hyperventilation). Residual serum samples were collected at the People’s Hospital of Heichi from non-anticoagulant tubes after the hospital’s routine diagnostic workup and tested within one hour. Samples with incomplete clinical records, insufficient volume for testing, or severe interferences were excluded. Samples covered a broad range of CO₂ concentrations and clinical diagnoses, aligning with the reagent’s linear range and intended use.

S2 Table presents the baseline characteristics of the study population, in which gender distribution was balanced (female, 43.4%; male, 56.6%), and the mean age was 56.12 years. The study included patients with various clinical diagnoses, including cardiovascular disease (16.25%), diabetes (10.63%), pulmonary disease (10.94%), physical examination cases (10.63%), renal disease (18.13%), and tumor diseases (8.44%).

The study protocol was approved by the Ethical Committee of the People’s Hospital of Hechi (Approval No. KY [2024-097-01]). The study adhered to the ethical guidelines outlined in the “Ethical Review Measures for Life Science and Medical Research Involving Humans” (2023), the World Medical Association (WMA) Declaration of Helsinki (2016), the International Ethical Guidelines for Health-related Research Involving Humans (2016), and Good Clinical Practice (GCP) principles. The study involved minimal risk to participants, as it used leftover samples from routine clinical diagnostic workup. All samples and data were anonymized by removing personal identifiers. Access to raw data was restricted, and there was no further follow-up of the participants. Informed consent was waived by the ethics committee due to the nature of the study.

### 2.2. Enzymatic assay principle

The Snibe TCO₂ assay measure total CO₂ through phosphoenolpyruvate carboxylase (PEPC) enzymatic method. PEPC catalyzes the reaction of HCO₃⁻ and phosphoenolpyruvate (PEP) to produce oxaloacetic acid and phosphoric acid in the presence of Mg^2+^. The oxaloacetic acid is then catalyzed by malate dehydrogenase (MDH) to produce malate. At the same time, the nicotinamide adenine dinucleotide (NADH) analog is oxidized to NAD+ analog. The consumption of NADH analogs results in a decrease in absorbance at 405 nm proportional to the concentration of CO₂ in the sample. 

### 2.3. Reagent and apparatus

Total CO₂ concentrations were measured using two enzymatic reagent kits. The Roche CO₂ assay (Bicarbonate Liquid [CO₂-L] kit) was performed on the c311 and c702 analyzers (Roche Diagnostics, Shanghai, China). The Snibe CO₂ assay (Bicarbonate [PEPC Enzymatic] kit) was conducted on the Biossays C8 and LABOSPECT 008 AS analyzers (Snibe Diagnostic, Shenzhen, China). Method comparison and onboard stability were assessed by comparing Snibe CO₂ on the Biossays C8 with Roche CO₂ on the c702. Accuracy was evaluated using Snibe CO₂ on the Biossays C8 and Roche CO₂ on the c311. Linearity and sample stability were examined using Snibe CO₂ on the Biossays C8. All tests were performed according to the manufacturers’ instructions, with calibration and quality control conducted per standard laboratory protocols to ensure accuracy and reliability.

### 2.4. Sample preservation conditions

To assess CO₂ stability, five serum samples with CO₂ concentrations of 20–30 mmol/L, which is around the reference interval, were stored in non-anticoagulant tubes and Eppendorf (EP) tubes at 2–8°C under uncapped conditions. CO₂ levels were measured using the Snibe CO₂ assay at 0, 0.5, 1, 2, 3, 4, 5, 17, 24, 40, and 48 hours. EP tubes contained serum extracted from non-anticoagulant tubes.

### 2.5. Statistical analysis

Continuous variables with a normal distribution are presented as means ± standard deviations, and those with a non-normal distribution as medians and interquartile ranges. Categorical variables are expressed as counts and percentages. Linearity of the Snibe CO₂ assay was assessed on day 0 and after 14 days onboard using least squares regression, with correlation determined by Pearson correlation coefficient (*r*) for both the Biossays C8 and LABOSPECT 008 AS platforms. Agreement between Snibe and Roche CO₂ assays was evaluated with Passing–Bablok regression and Bland-Altman analysis. Differences in CO₂ measurement results between Snibe and Roche assays in certain clinical diagnoses were tested using Wilcoxon signed-rank test. Serum CO₂ levels across clinical diagnoses for a certain assay were compared using Wilcoxon rank-sum test. Statistical analyses were performed with R, version 4.4.0, and *p* < 0.05 was considered significant.

## 3. Results

### 3.1. Accuracy

The performance of the Biossays C8 and cobas c311 platforms was evaluated using certified reference materials (CRM) from the National Institute of Metrology (NIM), China (GBW06101). Three levels were tested in duplicate on both platforms. The relative deviation from the target value ranged from 1.3% to −5.4% for Biossays C8 and from −2.2% to −6.4% for cobas c311 across the three levels (Table 1). The acceptance criteria for the bias of CO₂ assay was set to be ± 7.7%, the higher limit of the confidence interval for TCO₂ biological variation [18].

**Table 1:** Performance of two platforms (Biossays C8 and cobas c311) in testing certified reference materials (CRM).

Reference material	Target value (mmol/L)	Measured value (mmol/L)		Relative deviation		Acceptance criteria
		Snibe (n = 2)	Roche (n = 2)	Snibe (n = 2)	Roche (n = 2)	
Level 1	15.00	15.20	14.67	1.3%	−2.2%	±7.7%
Level 2	25.00	24.30	23.86	−2.8%	−4.6%	
Level 3	35.00	33.10	32.77	−5.4%	−6.4%	

### 3.2. Linearity

**Fig 1:**
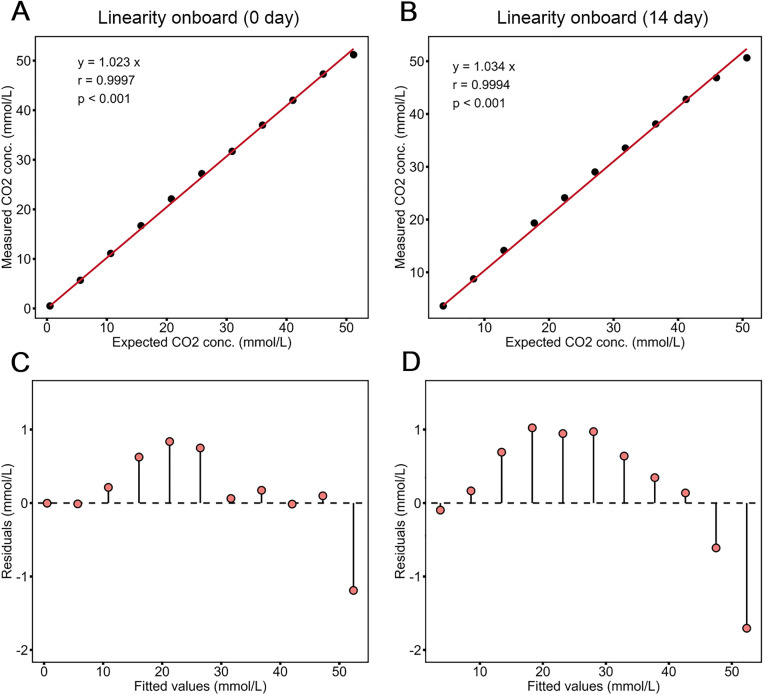
Linearity verification of Snibe CO₂ on Biossays C8. Linearity was evaluated on a) day 0 and b) after 14 days onboard. Linearity was assessed via least squares regression with intercept set to 0. c) and d) are the residual plots for the least square regression for day 0 and day 14, respectively. Correlation was analyzed through Pearson correlation coefficient. Each concentration was tested in triplicate.

The linearity of the CO₂ assay was evaluated using samples supplemented with sodium bicarbonate (Sangon Biotech, China) on day 0 and after 14 days onboard, following the CLSI EP06 guideline. An 11-step dilution series was prepared, with each dilution tested in triplicate. The resulting dilution curves demonstrated linearity up to a CO₂ concentration of at least 50 mmol/L. The assay exhibited excellent linearity on both day 0 and after 14 days onboard, with correlation coefficients of 0.9997 and 0.9994 for day 0 and day 14 respectively (Fig 1).

### 3.3. Calibration and onboard stability

We evaluated the calibration stability of the Snibe CO₂ assay by measuring two different control materials over a 14-day periods on two platforms: Biossays C8 and LABOSPECT 008 AS. Each time point was performed in duplicate, and the results remained within ±10% deviation throughout the observation period. The CO₂ calibration was found to be stable for 14 days (S1 Table). We also measured the two control materials in two different reagent lots and both lots passed the acceptance criteria (S5 Table).

**Fig 2:**
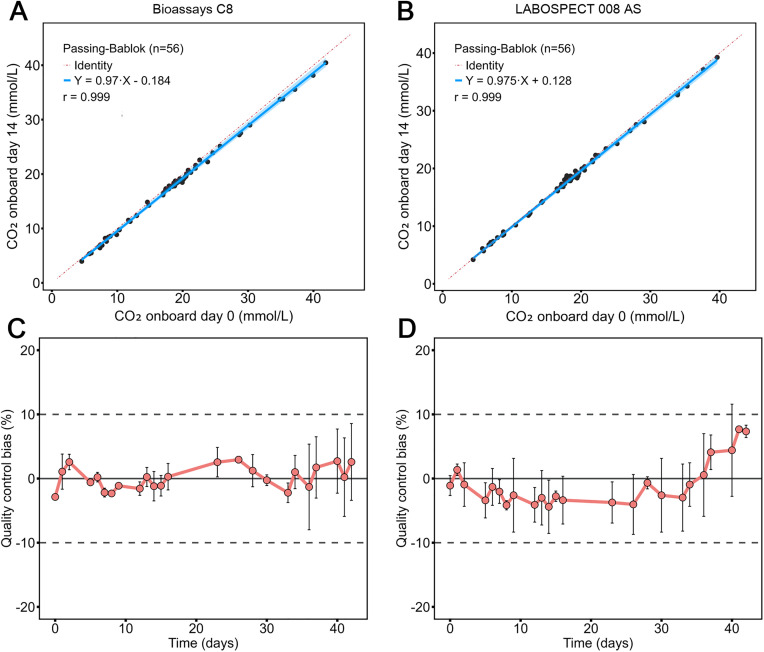
Calibration and onboard stability curves. Sample tests compared day 0 and day 14 onboard measurements on both platforms (A, B). Onboard stability was further evaluated for c) Snibe CO₂ and d) Roche CO₂ assays. The grey dashed line indicates ±10% deviation, which is the selected acceptance criteria. The sample size for each time point in the onboard stability study is 2. Quality controls were run in parallel with the experiments every time.

To evaluate onboard reagent stability, the same set of samples were tested on day 0 and day 14 on both platforms with the reagent kept onboard in these 14 days. A total of 56 samples with concentrations up to 50 mmol/L were tested once in each run. On the Bioassays C8 platform, a strong agreement was observed between day 0 and day 14 measurements, with a regression equation of y = 0.97x − 0.184 and a correlation coefficient of 0.999 (Fig 2A). Similar results were observed on the LABOSPECT 008 AS platform (Fig 2B).

As routine laboratory testing usually keeps reagents onboard with them stored uncapped, to further assess the real-world performance of this assay, we conducted evaluations in a clinical setting with reagents stored uncapped onboard. On the Biossays C8 analyzer, the Snibe CO₂ assay demonstrated excellent onboard stability. With uncapped reagent stored on the system, the assay maintained stability for 42 days without requiring recalibration, with quality control material measurements maintaining almost entirely within ±5% deviation from the target (Fig 2C). Similarly, the Roche CO₂ reagent on the Cobas c702 system demonstrated stability over 42 days, with relatively larger deviations (Fig 2D). The acceptance criteria for stability was ± 10% bias.

### 3.4. Method comparison

According to the manufacturer’s instructions for use, the linear ranges of both assays were 2–50 mmol/L, and the reference range was 22–29 mmol/L. The analysis was performed across different concentration categories (S3 Table), with balance largely preserved across categories. For example, in the 23–29 mmol/L range (based on Roche measurements), the proportion of samples within this range was 43.13% (138/320) for Snibe and 43.75% (140/320) for Roche, indicating no significant difference between the two assays (p = 1.000).

**Fig 3:**
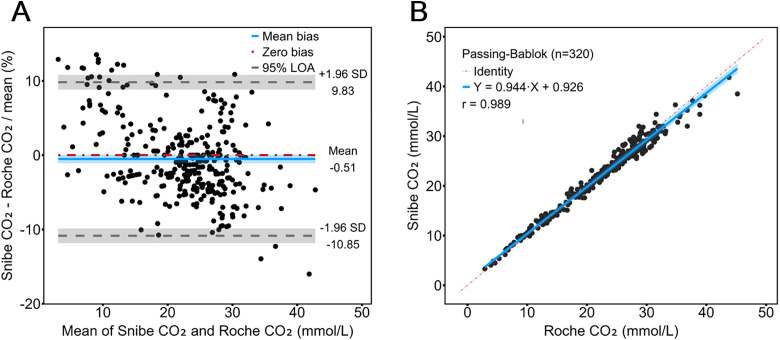
Comparison of serum CO₂ concentrations between Snibe and Roche assays. Bland-Altman plot (A) and Passing-Bablok regression (B) for the comparison between Snibe and Roche CO₂ assays. The sample size is 320 for both Bland-Altman and Passing-Bablok analyses. Abbreviations: SD, standard deviation; r, Pearson correlation coefficient.

The performance of the CO₂ assays was compared using 320 serum specimens across a concentration range of 2–50 mmol/L, with results obtained from both the Snibe and Roche CO₂ assays. Agreement between the methods was first assessed using the Bland-Altman analysis (Fig 3A), which showed a mean difference of −0.51% (95% limits of agreement, −10.85% to 9.83%) for the Snibe CO₂ assay compared with the Roche CO₂ assay. The relationship between CO₂ values measured by the two assays was further evaluated using Passing-Bablok regression (Fig 3B), yielding a Pearson correlation coefficient (*r*) of 0.989 and a regression equation of y = 0.944x + 0.926.

### 3.5. Assay comparison across different clinical diagnoses

The study included patients with various clinical diagnoses of diabetes (n = 34), pulmonary diseases (n = 35), tumor diseases (n = 27), cardiovascular diseases (n = 52), renal diseases (n = 58), physical examination (n = 34) and others (n = 80). The Healthy group consists of individuals undergoing routine physical examinations. The Pulmonary group includes patients with conditions such as pulmonary infections, pneumonia, chronic obstructive pulmonary disease (COPD), tuberculosis, respiratory failure, and asthma. The Renal group comprises individuals diagnosed with nephrotic syndrome, renal insufficiency, uremia, and chronic renal failure. The Tumor group includes patients with malignancies such as lung, cervical, rectal, and liver cancer. The Cardiovascular (CV) group consists of individuals with coronary atherosclerotic heart disease, hypertension, heart failure, and impaired cardiac function. The Others group includes patients with hyperthyroidism, anemia, arthritis, and lymphoma.

**Fig 4:**
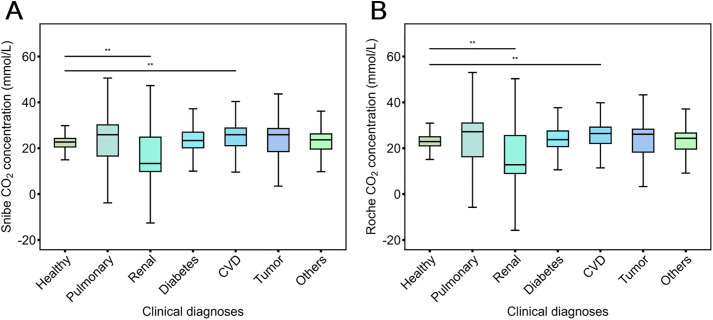
The same difference pattern of CO₂ levels in various disease groups versus healthy group measured with a) Snibe assay and b) Roche assay. Sample size: diabetes (n = 34), pulmonary diseases (n = 35), tumor diseases (n = 27), cardiovascular diseases (n = 52), renal diseases (n = 58), physical examination (n = 34) and others (n = 80). ** means p < 0.01.

The CO₂ concentrations in various clinical diagnosis groups were compared to the healthy group using Wilcoxon rank-sum test. The Snibe and Roche CO₂ assays demonstrated the same pattern in the statistical difference across all groups versus healthy group. For the Snibe assay, a statistically significant difference (p < 0.01; Fig 4A) was observed for the median CO₂ measurements between the renal group and the healthy group, which were 13.35 (9.87–24.85) versus 22.70 (20.55–24.28) mmol/L. Significantly increased measurements was observed in the cardiovascular disease group, with a significant difference (p < 0.01; Fig 4A). No significant differences were observed in the other groups. The Roche CO₂ assay showed the same trend (Fig 4B).

### 3.6. Effect of tube type

**Fig 5:**
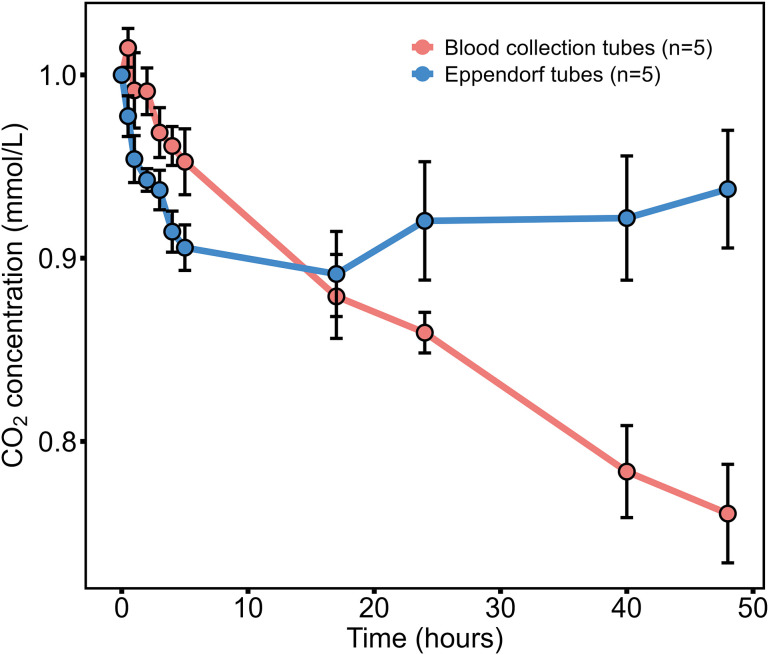
Sample stability across different tube types. Measurements were performed to evaluate CO₂ concentration changes under different storage conditions and assess sample stability over time. “Blood collection tube” refers to non-anticoagulant tubes.

Non-anticoagulant blood collection tubes and Eppendorf centrifuge tubes showed distinct CO₂ concentration change patterns. In non-anticoagulant tubes, CO₂ levels decreased progressively, with reductions of 4.7% at 5 hours, 14.1% at 24 hours, and 24.0% at 48 hours (Fig 5). In contrast, in EP tubes, the CO₂ concentration exhibited a more stable decline, with reductions of 9.4% at 5 hours, 8.0% at 24 hours, and 6.2% at 48 hours, showing less fluctuation over time (Fig 5).

## 4. Discussion

Serum total CO₂ concentration was measured using a coupled assay involving phosphoenolpyruvate carboxylase (PEPCase) and malate dehydrogenase (MDH), with the oxidation of nicotinamide adenine dinucleotide (NADH) monitored at 405 nm. The decrease in NADH concentration is directly proportional to the serum total CO₂ concentration, allowing for accurate measurement of serum total CO₂ levels [19]. Several studies have shown that serum total CO₂ concentration is closely correlated with bicarbonate (HCO₃⁻) concentration [20,21]. Enzymatic assays for total carbon dioxide offer advantages in clinical settings; however, reagent stability remains a challenge. Fluctuations in atmospheric CO₂ can affect measurements, leading to frequent quality control (QC) errors and the need for repeated recalibration [22]. Atmospheric CO₂ can impact the NADH oxidation process in reagent systems by altering enzyme activity and the reaction equilibrium [23,24]. These changes can cause unstable NADH oxidation rates, leading to quality control errors and the need for frequent recalibration of instruments. This phenomenon is particularly significant in assays involving PEPCase and MDH, highlighting the importance of controlling and stabilizing CO₂ concentrations during testing to ensure accurate results.

Unlike other enzymatic assays, which employ sealed packaging to indirectly maintain reagent stability, the Snibe assay resorts to developing a reagent system inherently more resistant to ambient CO₂. Based on the properties of PEPCase, the storage pH and the amount of PEP and PEPCase used in the reagents are optimized via inhibiting PEPCase for a minimal degradation rate of PEP during storage. Meanwhile, activation reagent is added before testing to reactivate the PEPCase, ensuring optimal analytical sensitivity. Optimization of the reaction system allowed the Snibe CO₂ assay to maintain accuracy for 42 days without recalibration, with control material deviations within ±5% (Fig 2C). In contrast, the Roche CO₂ assay on the Cobas c702 system showed relatively larger deviations over 42 days (Fig 2D). The improved reagent stability of the Snibe CO₂ assay could help clinical laboratories decrease the burden of storage regulation and the risk of retest due to reagent degradation. Improved reagent stability could also reduce waste from inventory expiry, enhancing cost efficiency. Higher operational efficiency could be another benefit as stable reagents require less frequent maintenance and are easier to be implemented in large-batch processing.

In addition to superior reagent stability, the Snibe CO₂ assay also showed smaller bias from the target than the Roche assay when tested with certified reference materials (Table 1). No significant differences were observed between the Snibe and Roche assays across concentration categories (S3 Table), with a Pearson correlation coefficient of 0.989, a regression equation of y = 0.944x + 0.926, and a Bland-Altman mean difference of −0.51% (Fig 3). The slightly lower regression ratio may reflect variability in certified reference materials. The Snibe and Roche assays also exhibited the same trend in CO₂ measurements across various diagnosis groups, with only the renal and CVD groups showing statistically significant difference from the healthy group (Fig 4).

Previous studies have highlighted discrepancies between local and central laboratory measurements, with CO₂ loss potentially contributing to reduced total CO₂ concentrations [25,26]. Zazra et al. reported a CO₂ loss rate of 2.5 mEq/L per hour from an open autoanalyzer cup [26], while Kirschbaum et al. observed a 4–5 mmol/L difference in total CO₂ measurements between local and central laboratories [25]. In this study, we found a paradoxical decline in CO₂ concentrations in both non-anticoagulant and EP tubes. Uncapped non-anticoagulant tubes showed a 24.0% decrease in CO₂, while EP tubes demonstrated a more stable 10.0% decline over 48 hours (Fig 5). Uncapped samples were investigated in this study as local hospitals typically leave their CO₂ sample tubes uncapped, which may differ elsewhere. Carbonic anhydrase (CA), which catalyzes the hydration of CO₂ to bicarbonate, appears to play a role in this process [27]. These discrepancies in CO₂ measurements could lead to misdiagnosis of acid-base disorders and unnecessary treatments with bicarbonate or other alkaline solutions. Consequently, the storage and handling conditions of blood samples after arrival in the laboratory can significantly affect the accuracy of subsequent analyses. Laboratories should define assay stability and implement procedures to minimize changes in this electrolyte.

The limitations of this study primarily come from its single center, single population nature. No external validation was conducted in this study and there was a limited coverage of diseases and methods. In this study, a total coincidence rate of 96.25% was observed at the diagnostic threshold of 22 mmol/L for acidosis between the Snibe and the Roche CO₂ assays, indicating a relatively small risk of misclassification. However, as acidosis is a lethal condition especially if misdiagnosed, multi-center or global studies are needed to validate the agreement between laboratory testing methods across different populations under real-world conditions. In addition, interference factors, such as paraproteins (including M proteins), hypertriglyceridemia, and endogenous antibodies, can lead to pseudohypobicarbonatemia [28, 29]. These factors were not investigated here and should be included in further studies to assess their potential impact on the Snibe CO₂ assay, particularly in cases where discrepancies occur between chemistry analyzers and bicarbonate calculations from blood gas analyzers. As we only tested one reagent lot in the stability studies, further research should be done on lot-to-lot difference in the stability. The effect of temperature cycling on the performance of the Snibe CO₂ assay could also be investigated in future studies. The influence of carbonic anhydrase on sample stability should also be examined. Efforts to enhance the onboard stability of CO₂ reagents will improve the accuracy of bicarbonate testing and contribute to more precise diagnoses and treatment decisions.

## 5. Conclusion

Accurate measurement of serum total CO₂ is essential for evaluating acid-base status in clinical practice. This study confirms the close correlation between Snibe CO₂ assay and Roche CO₂ assay and highlights the challenges posed by reagent stability and preanalytical factors. The Snibe CO₂ assay demonstrated superior stability and precision, maintaining accuracy for 42 days without recalibration, with lower deviations from target values compared to the Roche CO₂ reagent. The observed discrepancies in CO₂ measurements due to preanalytical factors, particularly CO₂ loss in uncapped tubes, underscore the need for standardized handling procedures to minimize errors in acid-base assessment.

## Supporting information

S1 Table The calibration stability of the Snibe CO₂ assay at onboard days 0, 1, 7, and 14. (DOCX)

S2 Table Demographic and clinical characteristics of individuals included in the study. (DOCX)

S3 Table Concentration category of samples included in the study. (DOCX)

S4 Table Assay comparison across different clinical diagnoses. (DOCX)

S5 Table Quality control measurement in 2 different reagent lots of the Snibe CO₂ assay. (DOCX)

## References

[1] Crystal GJ. Carbon dioxide and the heart: physiology and clinical implications. Anesth Analg. 2015;121(3):610–23. doi: 10.1213/ANE.0000000000000820 26287294

[2] Geers C, Gros G. Carbon dioxide transport and carbonic anhydrase in blood and muscle. Physiol Rev. 2000;80(2):681–715. doi: 10.1152/physrev.2000.80.2.681 10747205

[3] Hsia CC. Respiratory function of hemoglobin. N Engl J Med. 1998;338(4):239–47. doi: 10.1056/NEJM199801223380407 9435331

[4] Morikawa MJ, Ganesh PR. Acid-base interpretation: a practical approach. Am Fam Phys. 2025;111(1):148–55. 39964926

[5] Quade BN, Parker MD, Occhipinti R. The therapeutic importance of acid-base balance. Biochem Pharmacol. 2021;183:114278. doi: 10.1016/j.bcp.2020.114278 33039418 PMC7544731

[6] Huttmann SE, Windisch W, Storre JH. Techniques for the measurement and monitoring of carbon dioxide in the blood. Ann Am Thorac Soc. 2014;11(4):645–52. doi: 10.1513/AnnalsATS.201311-387FR 24701974

[7] Ungerer JP, Ungerer MJ, Vermaak WJ. Discordance between measured and calculated total carbon dioxide. Clin Chem. 1990;36(12):2093–6. doi: 10.1093/clinchem/36.12.2093 2123751

[8] Pippalapalli J, Lumb AB. The respiratory system and acid-base disorders. BJA Educ. 2023;23(6):221–8. doi: 10.1016/j.bjae.2023.03.002 37223696 PMC10201398

[9] Do C, Vasquez PC, Soleimani M. Metabolic alkalosis pathogenesis, diagnosis, and treatment: core curriculum 2022. Am J Kidney Dis. 2022;80(4):536–51. doi: 10.1053/j.ajkd.2021.12.016 35525634 PMC10947768

[10] Palmer BF, Clegg DJ. Respiratory acidosis and respiratory alkalosis: core curriculum 2023. Am J Kidney Dis. 2023;82(3):347–59. doi: 10.1053/j.ajkd.2023.02.004 37341662

[11] Halperin ML, Bear RA, Hannaford MC, Goldstein MB. Selected aspects of the pathophysiology of metabolic acidosis in diabetes mellitus. Diabetes. 1981;30(9):781–7. doi: 10.2337/diab.30.9.781 6790325

[12] Bonner R, Hladik G. Renal tubular acidosis: core curriculum 2025. Am J Kidney Dis. 2025;85(4):501–12. doi: 10.1053/j.ajkd.2024.08.014 39864011

[13] International Federation of Clinical Chemistry and Laboratory Medicine. IFCC Scientific Division, Working Group on Selective Electrodes. IFCC reference measurement procedure for substance concentration determination of total carbon dioxide in blood, plasma or serum. International federation of clinical chemistry and laboratory medicine. Clin Chem Lab Med. 2001;39(3):283–8. doi: 10.1515/cclm.2001.39.3.283 11350028

[14] Dimeski G, Badrick T, John AS. Ion Selective Electrodes (ISEs) and interferences--a review. Clin Chim Acta. 2010;411(5–6):309–17. doi: 10.1016/j.cca.2009.12.005 20004654

[15] Scott WJ, Chapoteau E, Kumar A. Ion-selective membrane electrode for rapid automated determinations of total carbon dioxide. Clin Chem. 1986;32:137–41. 3079679

[16] Frezzotti A, Margarucci Gambini AM, Coppa G, De Sio G. Total carbon dioxide measured by the Vitros enzymatic method. Clin Chem Lab Med. 1998;36(1):43–6. doi: 10.1515/CCLM.1998.008 9594085

[17] Chittamma A, Vanavanan S. Comparative study of calculated and measured total carbon dioxide. Clin Chem Lab Med. 2008;46(1):15–7. doi: 10.1515/CCLM.2008.005 17663632

[18] EFLM database. [Accessed 2025 August 11]. https://biologicalvariation.eu/meta_calculations

[19] Dobson GP, Veech RL, Hoeger U, Passonneau JV. Enzymatic determination of total CO2 in freeze-clamped animal tissues and plasma. Anal Biochem. 1991;195(2):232–7. doi: 10.1016/0003-2697(91)90322-k 1750672

[20] Hirai K, Ookawara S, Morino J, Minato S, Kaneko S, Yanai K, et al. Relationship between serum total carbon dioxide concentration and bicarbonate concentration in patients undergoing hemodialysis. Kidney Res Clin Pract. 2020;39(4):441–50. doi: 10.23876/j.krcp.19.126 32868493 PMC7770998

[21] Hirai K, Minato S, Kaneko S, Yanai K, Ishii H, Kitano T, et al. Approximation of bicarbonate concentration using serum total carbon dioxide concentration in patients with non-dialysis chronic kidney disease. Kidney Res Clin Pract. 2019;38(3):326–35. doi: 10.23876/j.krcp.19.027 31378012 PMC6727891

[22] Cuykx M, Develter M, Verschaeren J, Leenaerts D, Willemse J, Boes J. Quality control of total carbon dioxide (CO2) in serum or plasma using the Abbott Architect is affected by environmental pCO2 concentrations. Ann Clin Biochem. 2023;60(1):46–53. doi: 10.1177/00045632221128680 36085564

[23] Meyer CR, Rustin P, Wedding RT. A simple and accurate spectrophotometric assay for phosphoenolpyruvate carboxylase activity. Plant Physiol. 1988;86(2):325–8. doi: 10.1104/pp.86.2.325 16665904 PMC1054479

[24] Smith AM, Hylton CM, Rawsthorne S. Interference by phosphatases in the spectrophotometric assay for phosphoenolpyruvate carboxylase. Plant Physiol. 1989;89(3):982–5. doi: 10.1104/pp.89.3.982 16666652 PMC1055954

[25] Kirschbaum B. Spurious metabolic acidosis in hemodialysis patients. Am J Kidney Dis. 2000;35(6):1068–71. doi: 10.1016/s0272-6386(00)70041-2 10845818

[26] Bray SH, Tung RL, Jones ER. The magnitude of metabolic acidosis is dependent on differences in bicarbonate assays. Am J Kidney Dis. 1996;28(5):700–3. doi: 10.1016/s0272-6386(96)90251-6 9158207

[27] Occhipinti R, Boron WF. Role of carbonic anhydrases and inhibitors in acid-base physiology: insights from mathematical modeling. Int J Mol Sci. 2019;20(15):3841. doi: 10.3390/ijms20153841 31390837 PMC6695913

[28] Goldwasser P, Manjappa NG, Luhrs CA, Barth RH. Pseudohypobicarbonatemia caused by an endogenous assay interferent: a new entity. Am J Kidney Dis. 2011;58(4):617–20. doi: 10.1053/j.ajkd.2011.06.003 21849226

[29] Ma L, Zhao Z, Racine-Brzostek SE, Yang HS. A rare case of persistent pseudohypobicarbonatemia arising from chemistry analyzer-specific interference. Clin Chim Acta. 2021;519:308–10. doi: 10.1016/j.cca.2021.05.025 34051269

