# Supplementary material for: Stability, accuracy, and clinical performance of enzymatic total CO₂ measurement: Evaluation of the Snibe and Roche assays
Source: PLoS One. 2025 Oct 10;20(10):e0334228. doi: 10.1371/journal.pone.0334228 (PMC12513603; doi:10.1371/journal.pone.0334228)
Supplement: S1 Table — (DOCX) [file pone.0334228.s001.docx]

S1 Table. The calibration stability of the Snibe CO₂ assay at onboard days 0, 1, 7, and 14.

| Days | LABOSPECT 008 AS | | | | Biossays C8 | | | |
| --- | --- | --- | --- | --- | --- | --- | --- | --- |
|  | Control 1 | Deviation | Control 2 | Deviation | Control 1 | Deviation | Control 2 | Deviation |
| 0 | 15.09 | 2.62% | 32.59 | -0.34% | 14.89 | 1.29% | 32.55 | -0.45% |
| 1 | 15.37 | 4.56% | 32.30 | -1.21% | 14.42 | -1.93% | 32.19 | -1.55% |
| 7 | 15.71 | 6.89% | 32.14 | -1.70% | 15.34 | 4.33% | 32.49 | -0.65% |
| 14 | 15.52 | 5.58% | 31.33 | -4.18% | 15.70 | 6.80% | 31.91 | -2.42% |
