# Supplementary material for: Stability, accuracy, and clinical performance of enzymatic total CO₂ measurement: Evaluation of the Snibe and Roche assays
Source: PLoS One. 2025 Oct 10;20(10):e0334228. doi: 10.1371/journal.pone.0334228 (PMC12513603; doi:10.1371/journal.pone.0334228)
Supplement: S2 Table — (DOCX) [file pone.0334228.s002.docx]

S2 Table. Demographic and clinical characteristics of individuals included in the study.

| **Category** | **n = 320**^1^ |
| --- | --- |
| Gender |  |
| Female | 43.44% (139/320) |
| Male | 56.56% (181/320) |
| Age (years) |  |
| Mean (SD) | 56.12 (18.29) |
| Clinical disgnosis |  |
| Diabetes | 10.63% (34/320) |
| Pulmonary disease | 10.94% (35/320) |
| Tumor diseases | 8.44% (27/320) |
| Cardiovascular disease | 16.23% (52/320) |
| Renal disease | 18.13% (58/320) |
| Others | 25.00% (80/320) |
| Pysical examination | 10.63% (34/320) |

^1^% (n/N)
