# Supplementary material for: Stability, accuracy, and clinical performance of enzymatic total CO₂ measurement: Evaluation of the Snibe and Roche assays
Source: PLoS One. 2025 Oct 10;20(10):e0334228. doi: 10.1371/journal.pone.0334228 (PMC12513603; doi:10.1371/journal.pone.0334228)
Supplement: S3 Table — (DOCX) [file pone.0334228.s003.docx]

S3 Table. Concentration category of samples included in the study.

| Concentration category  (mmol/L) | n | | Percent | | | p value |
| --- | --- | --- | --- | --- | --- | --- |
|  | Snibe | Roche | | Snibe | Roche |  |
| 2-10 | 30 | 33 | | 9.38% | 10.31% | 0.808 |
| 11-15 | 38 | 34 | | 11.88% | 10.63% | 0.733 |
| 16-22 | 82 | 76 | | 25.63% | 23.75% | 0.847 |
| 23-29 | 138 | 140 | | 43.13% | 43.75% | 1.000 |
| 30-50 | 32 | 37 | | 10.00% | 11.56% | 0.616 |
| Total | 320 | 320 | | 100.0% | 100.0% | / |

The Chi-square test was used to calculate the p-value for testing the statistical difference between Snibe and Roche in each concentration category.
