# Supplementary material for: Stability, accuracy, and clinical performance of enzymatic total CO₂ measurement: Evaluation of the Snibe and Roche assays
Source: PLoS One. 2025 Oct 10;20(10):e0334228. doi: 10.1371/journal.pone.0334228 (PMC12513603; doi:10.1371/journal.pone.0334228)
Supplement: S4 Table — (DOCX) [file pone.0334228.s004.docx]

S4 Table. Assay comparison across different clinical diagnoses.

| Clinical diagnosis | n | Snibe (mmol/L) | Roche (mmol/L) |
| --- | --- | --- | --- |
| Diabetes | 34 | 23.35 (20.20-27.00) | 23.75 (20.75-27.53) |
| Pulmonary disease | 35 | 25.90 (16.60-30.20) | 27.20 (16.30-31.00) |
| Tumor diseases | 27 | 25.90 (18.55-28.60) | 26.10 (18.30-28.30) |
| Cardiovascular disease | 52 | 25.90 (21.13-28.83) | 26.40 (22.10-29.20) |
| Renal disease | 58 | 13.35 (9.87-24.85) | 12.80 (9.00-25.53) |
| Others | 80 | 23.65 (19.65-26.25) | 24.35 (19.63-26.63) |
| Healthy | 34 | 22.70 (20.55-24.28) | 22.90 (21.05-25.00) |

Median (1^st^ quartile-3^rd^ quartile) were used to statistically describe the data obtained by Snibe and Roche assays. The others group includes patients with hyperthyroidism, anemia, arthritis, and lymphoma. Wilcoxon signed-rank test was employed to calculate the p value.
