# Supplementary material for: Stability, accuracy, and clinical performance of enzymatic total CO₂ measurement: Evaluation of the Snibe and Roche assays
Source: PLoS One. 2025 Oct 10;20(10):e0334228. doi: 10.1371/journal.pone.0334228 (PMC12513603; doi:10.1371/journal.pone.0334228)
Supplement: S5 Table — (DOCX) [file pone.0334228.s005.docx]

S5 Table. Quality control measurement in 2 different reagent lots of the Snibe CO₂ assay.

| Control material | Target value | Reagent lot 1 | | Reagent lot 2 | | | Acceptance criteria |
| --- | --- | --- | --- | --- | --- | --- | --- |
|  |  | Repeat 1 | Repeat 2 | | Repeat 1 | Repeat 2 |  |
| QC1 | 14.7 | 1.29% | -0.54% | | 0.09% | 0.10% | ±7.7% |
| QC2 | 32.7 | -0.94% | -0.86% | | 1.63% | 0.13% |  |
